# EEG-based real-time diagnostic system with developed dynamic 2TEMD and dynamic ApEn algorithms

**DOI:** 10.3389/fphys.2023.1165450

**Published:** 2023-05-11

**Authors:** Ran Zhang, Linfeng Sui, Jinming Gong, Jianting Cao

**Affiliations:** ^1^ Saitama Institute of Technology, Saitama, Japan; ^2^ RIKEN Center for Advanced Intelligence Project (AIP), Tokyo, Japan

**Keywords:** EEG data analysis, dynamic, 2TEMD, ApEn, real time, diagnostic system

## Abstract

In real-time electroencephalography (EEG) analysis, the problem of observing dynamic changes and the problem of binary classification is a promising direction. EEG energy and complexity are important evaluation metrics in brain death determination in the field of EEG analysis. We developed two algorithms, dynamic turning tangent empirical mode decomposition to compute EEG energy and dynamic approximate entropy to compute EEG complexity for brain death determination. The developed algorithm is applied to analyze 50 EEG data of coma patients and 50 EEG data of brain death patients. The validity of the dynamic analysis is confirmed by the accuracy rate derived from the comparison with turning tangent empirical mode decomposition and approximate entropy algorithms. We evaluated the EEG data of three patients using the built diagnostic system. The experimental results visually showed that the EEG energy ratio was higher in a coma state than that in brain death, while the complexity was lower than that in brain death.

## 1 Introduction

Brain death is strictly defined as the complete, irreversible, and permanent loss of brain and brainstem function (Beecher, 1969). Previous research on the determination of brain death has focused on the field of clinical medicine. However, the clinical practice of brain death determination requires specialized physicians to perform a large number of rigorous compliance tests, including tests related to apnea tests that require the removal of the ventilator; there are certain risks in it and even cases of death have occurred (Marks and Zisfein, 1990). Therefore, it is crucial to use advanced signal-processing methods for electroencephalography (EEG) to provide reliable and objective scientific criteria that can support the clinical judgment in brain death determination.

Among the methods used to determine brain death from EEG signals, energy and complexity metrics are effective for EEG characterization and have shown excellent results in EEG signal classification, such as brain death determination (Shi et al., 2011) (Cao and Chen, 2008) and epilepsy detection (Sharma et al., 2018). To address these issues, empirical mode decomposition (EMD) (Huang et al., 1998), multivariate empirical mode decomposition (MEMD) (Rehman and Mandic, 2010) (Zahra et al., 2017), and turning tangent empirical mode decomposition (2TEMD) (Fleureau et al., 2010) of the intrinsic mode function (IMF) have been shown to be useful for extracting statistical features of EEG (Djemili et al., 2016). Some methods for analyzing complex signals have also been proposed for EEG analysis, complex empirical mode decomposition (CEMD) (Tanaka and Mandic, 2007), and improved eigenvalue decomposition of Hankel matrix–Hilbert transform (IEVDHM-HT) (Sharma and Pachori, 2018). 2TEMD shows the best overall performance among EMD, MEMD, and 2TEMD in processing EEG (Miao and Cao, 2017) and was more suitable for brain death determination. Complexity is a measure of the stochasticity of a time series. A higher complexity indicates greater randomness in the sequence. Approximate entropy (ApEn) (Pincus, 1991) is a stochastic complexity measure that describes the self-similarity of a time series in terms of patterns. It has been applied to EEG analyses, such as the analysis of sleep EEG data (Pincus, 1991). However, 2TEMD and ApEn cannot observe the changing process of EEG analysis. To address this limitation, dynamic 2TEMD and ApEn concepts are proposed (Miao and Cao, 2018).

In this paper, we have developed two algorithms, namely, D-2TEMD and D-ApEn, for the dynamic analysis of EEG signals. Comparing these algorithms with 2TEMD and ApEn algorithms, we have identified three key advantages: 1) Dynamic analysis can reduce noise and other effects, thereby improving the accuracy and reliability of the results. 2) Dynamic EEG analysis can help doctors observe changes in brain activity and assess and predict patients’ vital signs. 3) Real-time diagnosis systems based on dynamic algorithms can perform calculations while collecting EEG data, thereby saving time and reducing risk in patients. Based on D-2TEMD and D-ApEn algorithms, we have developed a real-time diagnostic aid system. We have demonstrated the efficacy of our system by performing binary classification of patients’ EEG as coma or brain death. Our analysis of EEG signals of a single patient involved analyzing the complexity and energy principles of EEG. We then applied the proposed system to analyze the EEG data of three patients obtained from the hospital. The patients comprised two typical cases in coma and brain death states and one special case from coma to brain death. Experimental results show that the overall trend in the distribution of EEG energy and complexity is evident in all three patients; although noise and other disturbances are present at a certain time point, it validated the effectiveness of the system and algorithm. Future studies can further validate the effectiveness of our system on a larger sample size. Additionally, there is a possibility of applying the system to other medical conditions.

## 2 D-2TEMD and D-ApEn algorithms

### 2.1 Developed D-2TEMD algorithm

**FIGURE 1:**
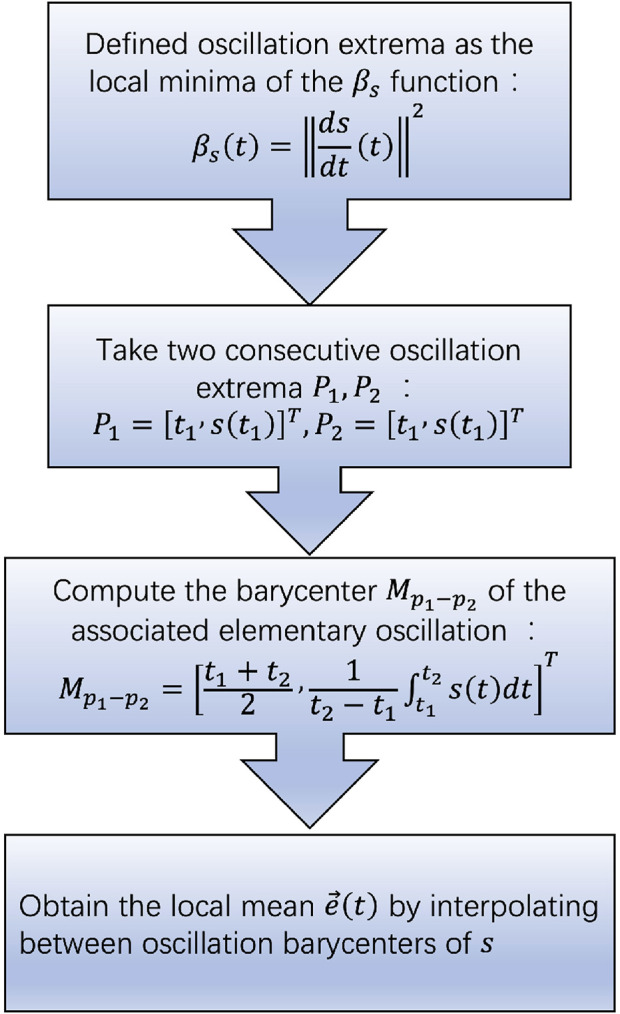
Flowchart of the computation of local mean for 2TEMD.

The 2TEMD algorithm is a signal decomposition method that improves upon EMD. It extracts intrinsic mode functions by redefining the average trend of the signal and calculating the average envelope through interpolation between local extremal points. The IMF is then extracted using tangent line iteration. This specific process is shown in Figure 1.

The signal *s* is decomposed into a finite set of IMFs $\sum_{n=1}^{N}IMF_{n}$ and a residual term, where IMFs represent local oscillatory components of the signal, and the residual term *r*(*t*) represents the signal’s long-term trend and high-frequency noise.

$$s\left(t\right)=\overset{n}{\underset{i=1}{\sum }}IMF_{i}+r\left(n\right).$$ (1)

We developed the D-2TEMD algorithm, which is the extended form of 2TEMD by introducing a time window, Δ*t*, which serves as a computation step in 2TEMD. For each Δ*t*, we computed IMFs and empirically removed the first four high-frequency residual signals (Miao et al., 2017). The remaining IMFs were then used to analyze the EEG data and obtain a dynamic distribution of EEG energy. EEG energy was defined as the product of the power specification value in the frequency band and the corresponding recording time. For a multivariate signal ${\vec{s}\left(k•\Delta t\right)}_{k=0}^{K}=\vec{s}\left(0•\Delta t\right),\vec{s}\left(1•\Delta t\right),\ldots ,\vec{s}\left(K•\Delta t\right)$ with n components from T1 to T2, the D-2TEMD process is as follows:

1) Constructing an auxiliary function: We initialize the number of iterations *j* = 1, the number of IMFs *i* = 1, and the number of time steps *k* = 0.

$${\vec{r}}_{i}\left(k\cdot \Delta t\right)={\left\{\vec{s}\left(k\cdot \Delta t\right)\right\}}_{k=0}^{K},{\vec{h}}_{i,j-1}\left(k\cdot \Delta t\right)={\vec{r}}_{i}\left(k\cdot \Delta t\right).$$ (2)

2) Identifying local extrema: We compute the barycenter $M_{{\left(i,j-1\right)}_{P_{k_{x}}}-P_{k_{x+1}}}\left(k\cdot \Delta t\right)$ of random consecutive oscillation extrema $P_{k_{x}}$ and $P_{k_{x+1}}$ in a period of (*k* ⋅Δ*t*).

$$M_{{\left(i,j-1\right)}_{P_{k_{x}}-P_{k_{x+1}}}}\left(k\cdot \Delta t\right)={\left[\frac{k_{x}\cdot \Delta t+k_{x+1}\cdot \Delta t}{2},\frac{1}{k_{x}\cdot \Delta t-k_{x+1}\cdot \Delta t}\int_{k_{x+1}\cdot \Delta t}^{k_{x}\cdot \Delta t}{\vec{h}}_{i,j-1}\left(t\right)dt\right]}^{T}.$$ (3)

3) Calculation of the mean envelope: The average signal trend is obtained by interpolating between the centers of oscillation of ${\vec{h}}_{i,j-1}\left(k\cdot \Delta t\right)$ .

$${\vec{e}}_{i,j-1}\left(k\cdot \Delta t\right).$$ (4)

4) Extracting IMFs: We subtract ${\vec{e}}_{i,j-1}\left(k\cdot \Delta t\right)$ from the given signal ${\vec{h}}_{i,j-1}\left(k\cdot \Delta t\right)$ and define ${\vec{h}}_{i,j}\left(k\cdot \Delta t\right)={\vec{h}}_{i,j-1}\left(k\cdot \Delta t\right)-{\vec{e}}_{i,j-1}\left(k\cdot \Delta t\right)$ . We obtain the following:

$${\vec{IMF}}_{i}\left(k\cdot \Delta t\right)={\vec{h}}_{i,j}\left(k\cdot \Delta t\right).$$ (5)

Otherwise, the iteration steps from (2) to (4)are repeated.

5) Iterating to extract IMFs: We define ${\vec{r}}_{i}\left(k\cdot \Delta t\right)={\vec{r}}_{i}\left(k\cdot \Delta t\right)-{\vec{IMF}}_{i}\left(k\cdot \Delta t\right)$ . If the result signal is monotonous, we can get the decomposition results of signals during *k* ⋅Δ*t*.

$$\vec{s}\left(k\cdot \Delta t\right)=\overset{N}{\underset{i=1}{\sum }}{\vec{IMF}}_{i}\left(k\cdot \Delta t\right)+{\vec{r}}_{N}\left(k\cdot \Delta t\right).$$ (6)

Otherwise, we repeat the iteration steps from (2) to (5).

6) We determine whether the elapsed time *k* ⋅Δ*t* exceeds the end time *T*_2_; if it does, then the process moves toward the end, and the final decomposition result is as follows:

$${\left\{\vec{s}\left(k\cdot \Delta t\right)\right\}}_{k=0}^{K}={\left\{\overset{N}{\underset{i=1}{\sum }}{\vec{IMF}}_{i}\left(k\cdot \Delta t\right)+{\vec{r}}_{N}\left(k\cdot \Delta t\right)\right\}}_{k=0}^{K}.$$ (7)

If it does not, we move the time window and repeat the steps from step 2 to step 6.

### 2.2 Developed D-ApEn algorithm

Complexity measures based on approximate entropy have shown good performance in the statistical characterization of EEG signals. High intensity and the absence of spontaneous brain activity from quasi-brain-death EEG can be obtained by power spectral pattern analysis. It appears to have potential applications in a wide variety of physiological and clinical time series data. ApEn is a measure of the regularity or predictability of a time series. The ApEn statistics of a time series measure the logarithmic probability that runs on patterns of length m that are close to each other and will also remain close in the next incremental comparison *m* + 1 (Pincus and Goldberger, 1994). A higher probability of remaining close (with high regularity) results in a lower ApEn value and *vice versa*. Approximate entropy analysis is only carried out for a segment of the data and does not reflect the approximate entropy change process for the entire measurement time.

We consider a sequence A(1,2 … ,N), for computing the ApEn (A, *m*, and *r*) (A: N-m+1, m: length of the vector series, and *r*: tolerance parameter) of that sequence, and let *D* (*i*, *j*) denote the distance between two vectors *v*(*i*) and *v*(*j*) (*i* and *j* ≤ N-m+1), which is defined as the maximum difference in scalar components of *v*(*i*) and *v*(*j*).

$$D\left[v\left(i\right),v\left(j\right)\right]=\max_{\text{k}=1,2,\ldots ,m}\left(\left|v\left(i+k-1\right)-v\left(j+k-1\right)\right|\right).$$ (8)

We define *C* ^*m*,*r*^(*i*), which is the probability of finding a vector that differs from v(*i*) by less than distance *r*. Also, *ϕ* ^*m*,*r*^, the natural logarithmic average over all the vectors of the *C* ^*m*,*r*^(*i*) probability is as follows:

$$C^{m,r}\left(i\right)=\frac{N^{m,r}\left(i\right)}{N-m+1},\;\phi^{m,r}=\frac{\sum_{i=1}^{N-m+1}\log C^{m,r}\left(i\right)}{N-m+1}.$$ (9)

For *m* + 1, we repeat the aforementioned steps and compute *ϕ* ^*m*+1,*r*^. The ApEn statistics is given by the following:

$$ApEn\left(A,m,r\right)=\phi^{m,r}-\phi^{m+1,r}.$$ (10)

ApEn is only evaluated for a specific segment of the signal and does not reflect the process of approximate entropy change. ApEn is susceptible to high-frequency electron interference present in real recorded EEG signals. Therefore, to ensure the accuracy and reliability of ApEn in full-frequency EEG signals, the D-ApEn algorithm is used, which is an extension of the existing ApEn algorithm by introducing a time variable Δ*t*, which extends the existing ApEn analysis method on the time axis to obtain the dynamic approximate entropy algorithm (Shi et al., 2010). It can reflect the dynamic complexity changes throughout the recording time and avoid the loss of detailed information. This specific algorithm is as follows:

For a time series A(Δ*t*) of time length T, (Δ*t* = 1, - - -, N) unit in seconds, in order to compute ApEn(A(Δ*t*), *m*, and *ɛ*),

$$A_{\Delta t}=\left[x_{\Delta t}\left(1\right),x_{\Delta t}\left(2\right),\ldots ,x_{\Delta t}\left(N\right)\right].$$ (11)

For this sequence, a vector sequence of length *m*, *v*_Δ*t*_(*i*) and *v*_Δ*t*_(*j*), is first constructed from the signal sample A (Δ*t*).

$$D\left(i,j\right)=\max_{\Delta t=1,\ldots ,m}\left|v_{l}\left(i\right)-v_{l}\left(j\right)\right|.$$ (12)

We let $D\left[v_{\Delta t}\left(i\right),v_{\Delta t}\left(j\right)\right]$ denote the distance between two vectors *v*_Δ*t*_(*i*) and *v*_Δ*t*_(*j*) (*i* and *j* ≤ *N* − *m* + 1), which is defined as the maximum difference between the scalar components of *v*_Δ*t*_(*i*) and *v*_Δ*t*_(*j*).

$$D\left[v_{\Delta t}\left(i\right),v_{\Delta t}\left(j\right)\right]=\underset{k=1,\ldots ,m}{\max},\ldots ,m\left(\left|v_{\Delta t}\left(i+k-1\right)-v_{\Delta t}\left(j+k-1\right)\right|\right).$$ (13)

For a given threshold *ɛ* and vector *v*_Δ*t*_(*i*), when *j* ≤ *N* − *m* + 1, the number of vectors *v*_Δ*t*_(*i*) whose distance from *v*_Δ*t*_(*j*) is less than *ɛ* is calculated, and then, $C_{i}^{m}\left(\varepsilon ,\Delta t\right)$ denotes the probability that the distance from *v*_Δ*t*_(*i*) is less than *ɛ* for all vectors *v*_Δ*t*_(*j*) when *j* ≤ *N* − *m* + 1, denoted by the following:

$$C_{i}^{m}\left(\varepsilon ,\Delta t\right)=\frac{D\left[v_{\Delta t}\left(i\right),v_{\Delta t}\left(j\right)\right]\leq r}{N-m+1},$$ (14)

$$\phi^{m}\left(\varepsilon ,\Delta t\right)=\frac{\sum_{i=1}^{N-m+1}\ln C_{i}^{m}\left(\varepsilon ,\Delta t\right)}{N-m+1}.$$ (15)

Repeating the aforementioned steps for *m* + 1 and computing *ϕ* ^*m*+1^ (*ɛ*, Δ*t*), D-ApEn is given as follows:

$$ApEn\left(m,\varepsilon ,\Delta t\right)=\phi^{m\left(\varepsilon ,\Delta t\right)}-\phi^{m+1\left(\varepsilon ,\Delta t\right)}.$$ (16)

### 2.3 EEG energy and complexity based on D-2TEMD and D-ApEn

**FIGURE 2:**
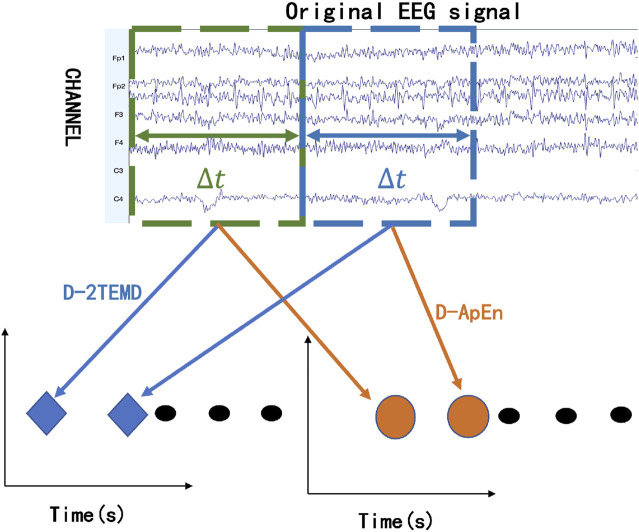
D-2TEMD and D-ApEn schematics.

The D-2TEMD algorithm introduces a time window of length Δ*t*, where Δ*t* is a controllable parameter, shown in Figure 2. In this experiment, Δ*t* is 1 s (i.e., 1000 sampling rate). For this time, we have designed a loop where one EEG with one Δ*t* step is processed and stored. Also, as the time window and time step were sliding, EEG data were analyzed by applying D-2TEMD and D-ApEn algorithms to obtain the dynamic EEG energy distribution and dynamic complexity distribution in the time domain.

**FIGURE 3:**
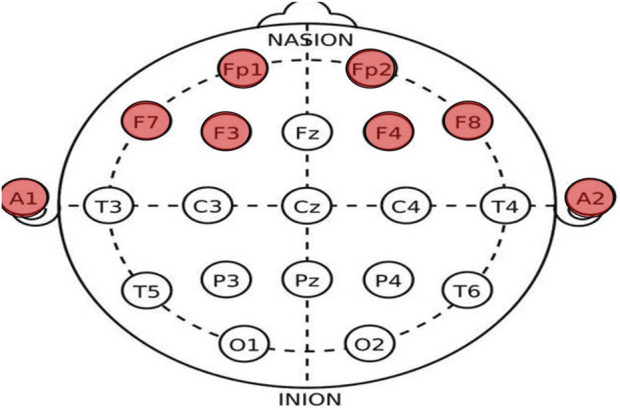
International 10–20 standard system electrode position distribution map (red-colored ones are the channels used in this experiment).

We compared brain death determination algorithms based on EEG energy and complexity. In the comparison, we randomly selected 140 EEG datasets from 36 cases to ensure data uniformity. These included 70 coma patients and 70 brain death patients, as diagnosed by medical professionals. We randomly selected 20 EEG datasets as a standard and used the remaining 100 EEG datasets to calculate energy and complexity using 2TEMD, ApEn, D-2TEMD, and D-ApEn methods to derive accuracy measures. The EEG data were collected at a hospital in Shanghai, China, between June 2004 and March 2006, with the permission of the patients’ families. A portable EEG system (NeuroScan ESI) was used and a total of nine electrodes were selected for the examination, six exploratory electrodes (Fp1, Fp2, F3, F4, F7, and F8) were placed on the forehead, and two electrodes (A1 and A2) were placed on the earlobes as a reference, as shown in Figure 3. The EEG sampling rate was 1000 Hz, and the electrode resistance was set to less than 8 kΩ. The EEG data were supervised by a neurologist and performed by medical personnel.

The 2TEMD algorithm calculates the average energy per second. m and r of the ApEn algorithm are 1000 and 1, respectively. Δ*t* for the dynamic analysis is 1 s. We analyzed the EEG data of 20 coma patients and found the EEG energy per second ranging from 1.8 × 10^4^ ∼ 1.8 × 10^5^, with a complexity of about 0.3. We also analyzed the EEG data of 20 patients in a quasi-brain-dead state and found the EEG energy per second ranging from 6.2 × 10^3^ ∼ 8 × 10^3^, with a complexity of about 1.1. We calculated the accuracy of the remaining 100 data points using the following criteria, as shown in Table 1.

**TABLE 1:** Comparison of four EEG brain death determination methods: 2TEMD, ApEn, D-2TEMD, and D-ApEn.

Author (year)	Method	Accuracy (%)
shi et al. (2011)	2TEMD	89
D-2TEMD	D-2TEMD	97
cao et al. (2008)	ApEn	92
D-ApEn	D-ApEn	94

Energy calculation using the 2TEMD algorithm, due to its reliance on a significant number of matrix operations, exhibits computational inefficiencies when processing longer time series data. Specifically, the processing time required to analyze 120 s of EEG data using the 2TEMD algorithm that exceeds 800 s, while the D-2TEMD algorithm requires a comparatively shorter processing time of approximately 500 s.

## 3 Implementation of the real-time diagnosis system

### 3.1 Hardware structure

**FIGURE 4:**
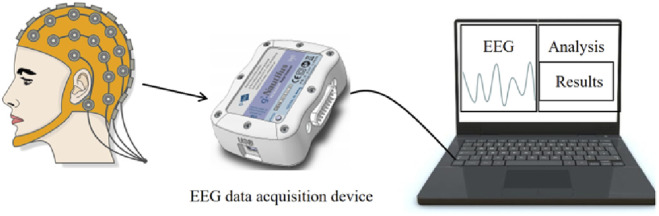
Real-time diagnostic system.

The system we designed consists of an EEG data acquisition device and a laptop computer. The system connections are shown in Figure 4. For the determination of brain death, the accuracy of EEG extraction is of utmost importance and the accuracy of EEG extraction is of paramount importance. Therefore, we use g.Nautilus as the experimental device. It has international standard electrode caps, more channels for us to choose from, and relatively higher measurement accuracy. Data acquisition and processing are performed in a laptop computer connected to the g.Nautilus electrode cap through the g.Nautilus unlimited-communication device.

### 3.2 System software design

**FIGURE 5:**
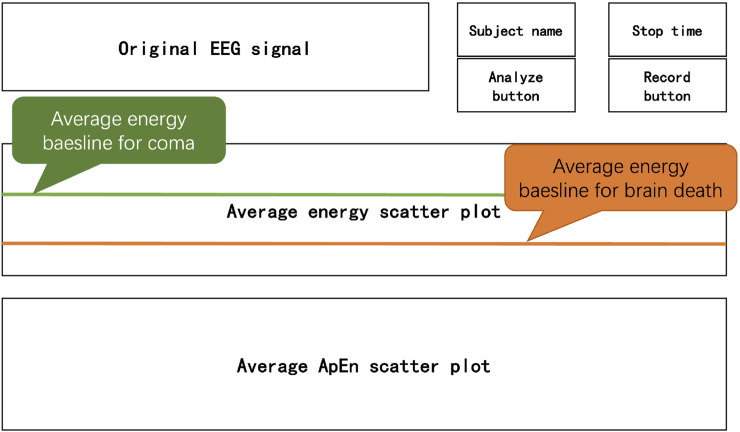
GUI for the real-time diagnostic system.

The GUI we designed for this diagnostic system is shown in Figure 5; the top right corner can define the subject’s name and time of this acquisition, the “Recording” button is used to open the system framework and start the acquisition of EEG, and we click the “Analysis” button to start the analysis and display of the EEG data. The upper axial plot shows the raw EEG signal for each channel. The middle plot dynamically displays the average energy of all channels based on the results of D-2TEMD calculations. Each point represents the average energy per unit time Δ*t*. The energy baselines for coma and brain death are set based on reference values calculated separately from the EEG data of 20 coma patients and 20 brain death ones, each obtained from the hospital. Finally, the axial plot at the bottom shows the average complexity of all channels, with each point representing the average complexity of Δ*t*.

## 4 Experiment results

The EEG data used to calculate the criteria for coma and brain death groups, with 20 patients each, showed an average energy analysis of 1.8 × 10^4^ and 4.96 × 10^3^ per second, respectively, for six channels, represented as two straight lines through the GUI window. The patients were divided into coma, brain death, and from coma to brain death groups. Each point of energy and complexity in the GUI represents the average energy and complexity of the EEG data for the six channels in 1 s. We considered the average energy converging to 1.8 × 10^4^ and the average complexity converging to 0.3 in the coma group, and the average energy converges to 4.96 × 10^3^ and the average complexity converges to 1.1 in the brain death group. The results of the three sets of experiments are as follows.

**FIGURE 6:**
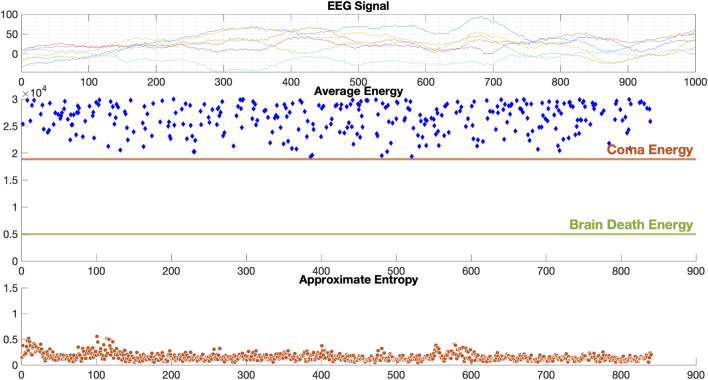
Results for coma patient EEG data.

**FIGURE 7:**
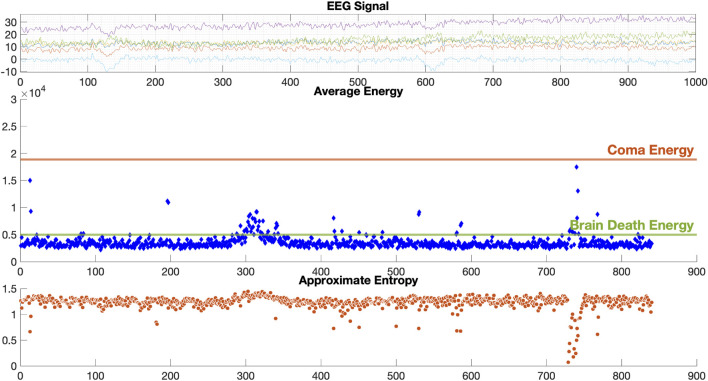
Results for brain death EEG data.

**FIGURE 8:**
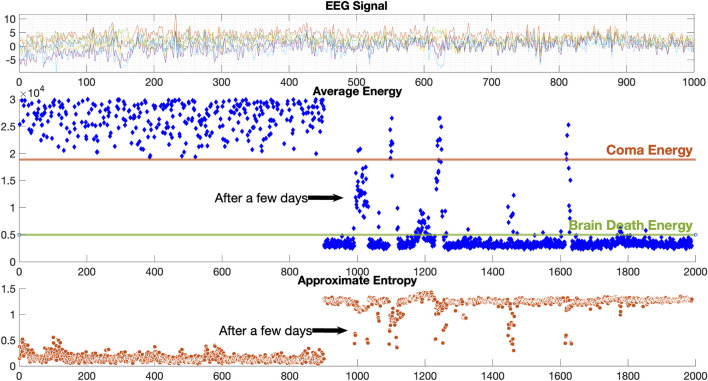
Results for coma turning to brain death EEG data.

The results of EEG analysis calculated by the diagnostic system are shown in Figure 6. GUI shows the original EEG data, the calculated EEG energy after D-2TEMD, and the distribution of EEG complexity calculated by D-ApEn, which shows the characteristics of EEG from all aspects and determines the EEG state. The top coordinate system is the original six-channel EEG signal, and the other two coordinate systems are the six-channel average energy and six-channel average complexity after classification by the binary classification method. The results of the analysis showed that the dynamic energy range of EEG of coma patients was 1.8 × 10^4^ ∼ 3 × 10^4^ and the complexity ranged from 0 to 0.5. This is consistent with our rules for determining the characteristics of the coma state. Figure 7 shows the results of the EEG analysis of a brain death patient. This result shows that the overall dynamic EEG energy in the brain death state is close to 4.96 × 10^3^, and the complexity distribution is between 1 and 1.5. This is consistent with our rules for determining the characteristics of the brain death state. Figure 8 shows the results of a particular EEG analysis. A few days earlier, the dynamic energy range of EEG of coma patients was 1.8 × 10^4^ ∼ 3 × 10^4^ and the complexity distribution was between 0 and 0.5. A few days later, the overall dynamic EEG energy in the brain death state is close to 4.96 × 10^3^ and the complexity distribution was between 1 and 1.5. This is consistent with the rules we used to determine the characteristics of the coma and brain death state. It is worth noting that the results of the analysis during brain death occasionally fluctuated based on finding cases where it was known that drug resuscitation was used at that time.

Experimental results show that the more pronounced the brain activity, the higher the EEG energy. Also, the lower the complexity, the lower the probability that the sequence will generate new patterns and it has a certain regularity and predictability. Therefore, it can be inferred that there was brain activity in the coma group, while there was almost no brain activity in the brain death group because the dynamic EEG energy distribution was significantly higher in the coma group than that in the brain death group. Furthermore, it can be inferred that brain activity rhythms were present in the EEG of the coma group, while almost disordered noise was present in the EEG of the brain death group because the complexity distribution was lower in the coma group than that in the brain death group. In addition, dynamic distribution can provide more accurate and reliable results compared to 2TEMD and ApEn. The dynamic analysis of the diagnostic system also provides a reference point for physicians by showing the process of changes in a patient’s physical characteristics. The diagnostic system may be less friendly to physicians who are not specialized in EEG signal processing, and we hope to generate a clearer and more understandable index to help physicians determine brain death in future studies.

## 5 Conclusion

In this paper, we develop new algorithms, D-2TEMD and D-ApEn, for the real-time EEG analysis, which can achieve EEG dynamic analyses by introducing time windows. Using the accuracy metric, they are shown to have a high degree of validity and robustness in calculating the energy and complexity changes of EEG. Our results show that D-2TEMD can easily discriminate coma and brain death patients based on the significant differences in EEG energy values. D-ApEn has an extremely high regularity and predictability, with a strong periodicity of intracerebral activity in coma patients and a complexity close to 0, a weak intracerebral activity in coma patients, mainly due to noise superposition, and a complexity close to 1 in brain-dead EEG. Our experiments further validate the effectiveness of D-2TEMD and D-ApEn algorithms, even if the results are affected by noise, etc., but the overall trend is clear. The system also includes the ability to classify other EEG signals, such as healthy subjects, sleep, and anesthesia, when sufficient sample data are available. The metrics of this system may be more complex for physicians who are not familiar with the EEG analysis. In the future, we will provide a simpler and more understandable index to assist physicians in determining brain death.

## Data Availability

The raw data supporting the conclusions of this article will be made available by the authors, without undue reservation.
